# Kolaviron, a *Garcinia* biflavonoid complex ameliorates hyperglycemia-mediated hepatic injury in rats via suppression of inflammatory responses

**DOI:** 10.1186/1472-6882-13-363

**Published:** 2013-12-20

**Authors:** Omolola R Ayepola, Novel N Chegou, Nicole L Brooks, Oluwafemi O Oguntibeju

**Affiliations:** 1 Department of Biomedical Sciences, Cape Peninsula, Oxidative Stress Research Centre, University of Technology, Bellville, South Africa; 2 Department of Wellness Sciences, Cape Peninsula University of Technology, Cape Town, South Africa; 3 Department of Biomedical Sciences, Centre of Excellence for Biomedical Tuberculosis Research and MRC Centre for Molecular and Cellular Biology, Division of Molecular Biology and Human Genetics, Stellenbosch University, Tygerberg, South Africa

**Keywords:** Diabetes, Hepatic injury, Kolaviron, Pro-inflammatory cytokine, Chemotactic protein

## Abstract

**Background:**

Chronic inflammation plays a crucial role in hyperglycemia-induced liver injury. Kolaviron (KV), a natural biflavonoid from *Garcinia kola* seeds have been shown to possess anti- inflammatory properties which has not been explored in diabetes. To our knowledge, this is the first study to investigate the effect of KV on pro-inflammatory proteins in the liver of diabetic rats.

**Methods:**

Diabetes was induced by a single intraperitoneal injection of streptozotocin (STZ) (50 mg/kg) in male Wistar rats. Kolaviron (100 mg/kg) was administered orally five times a week for six weeks. The concentrations of cytokines and chemokine were measured using Bio-plex Pro™ magnetic bead-based assays (Bio-Rad Laboratories, Hercules, USA). Plasma glucose and serum biomarkers of liver dysfunction were analyzed with diagnostic kits in an automated clinical chemistry analyzer. Insulin concentration was estimated by radioimmunoassay (RIA).

**Result:**

Kolaviron (100mg/kg) treatment significantly ameliorated hyperglycemia and liver dysfunction. Serum levels of hepatic marker enzymes were significantly reduced in kolaviron treated diabetic rats. Kolaviron prevented diabetes induced increase in the hepatic levels of proinflammatory cytokines; interleukin (IL)-1beta, IL-6, tumour necrosis factor (TNF-α) and monocyte chemotactic protein (MCP-1).

**Conclusion:**

The results of this study demonstrate that the hepatoprotective effects of kolaviron in diabetic rats may be partly associated with its modulating effect on inflammatory responses.

## Background

Type 1 diabetes mellitus (DM) is an autoimmune disorder involving immune mediated recognition of pancreatic β cells by auto-reactive T cells with subsequent release of pro- inflammatory cytokines that worsen the disease state [1]. DM characterized by prolonged hyperglycemia in the postprandial and/or fasting state [2] results from impaired insulin mediated glucose metabolism. Uncontrolled hyperglycemia leads to progressive development of microvascular and macrovascular complications, causing morbidity and mortality in diabetic patients [3-5]. Diabetes is associated with an increased risk of hepatic injury [6,7]. It has been reported that the standardized mortality rate from end-stage liver disease (i.e. cirrhosis) in diabetic patients is higher than those with cardiovascular disease [8].

To a large extent, the effect of hyperglycemia is mediated by an elevation in the levels of pro-inflammatory proteins. Over-production of several inflammatory mediators such as growth factors, pro-inflammatory cytokines and chemokines has been documented in DM [9-11].

Type 1 DM is considered as an inflammatory process in which a significant increase of cytokines was found in the blood of patients with this disease. The response of hepatocytes to pro-inflammatory cytokines promotes the expression of genes that mediate the inflammatory process [12]. Furthermore, increased oxidative stress and chronic inflammation affects insulin secretion and sensitivity [13]. Targeting inflammatory mediators signaling through the use of anti-inflammatory agents could therefore improve therapeutic options for diabetic liver disease, a diabetic complication that is gradually gaining recognition.

Bitter kola (*Garcinia kola*) belongs to the family of plants called Guttiferae and the genus *Garcinia. Garcinia kola* seeds have been shown to contain a complex mixture of polyphenolic compounds, biflavonoids, prenylated benzophenones and xanthones which account for the majority of its effects [14,15]. Kolaviron (KV) is an extract from the seeds of *Garcinia kola*, containing a complex mixture of biflavonoids and polyphenols [16]. A number of studies have confirmed the antioxidative and anti-inflammatory effects of kolaviron in chemically-induced toxicity, animal models of diseases and in cell culture [17-20]. Although the glucose lowering effect of kolaviron has been reported in animal models of diabetes mellitus [21,22], no study has addressed the effect of KV on inflammatory biomarkers in diabetes. In the present study, we investigated the effects of kolaviron in modulating inflammatory responses in the liver of streptozotocin-induced diabetic rats.

## Methods

### Plant materials

Fresh seeds of *Garcinia kola* were purchased from Bodija market in Ibadan, Oyo State, Nigeria and authenticated by Professor E. A Ayodele at the Department of Botany, University of Ibadan. A voucher specimen is available at the herbarium of the Forestry Research Institute of Nigeria (FRIN), Ibadan.

### Extraction of kolaviron

*Garcinia kola* seeds were peeled, sliced and air-dried (25–28°C). Kolaviron was isolated according to the method of Iwu *et al.*[20]. Briefly, the powdered seeds were extracted with light petroleum ether (bp 40–60°C) in a soxhlet for 24 hr. The defatted dried product was repacked and extracted with acetone. The extract was concentrated and diluted twice its volume with water and extracted with ethylacetate (6 × 300 ml). The concentrated ethylacetate yielded kolaviron, a golden yellow solid [16].

### Ethics statement

The study protocol was approved by the Faculty of Health and Wellness Sciences Research Ethics Committee of Cape Peninsula University of Technology (Ethics Certificate no: CPUT/HW-REC 2012/AO4). All the animals received humane care in accordance to the criteria outlined in the ‘Guide for the Care and Use of Laboratory Animals’ prepared by the National Academy of Science (NAS) and published by the National Institute of Health (Publication no. 80–23, revised 1978).

### Animals

Adult male Wistar rats, weighing about 240–290 g were housed in individual plastic cages at the animal facility of the Medical Research Council, South Africa. They were supplied with water and standard rat feed *ad libitum*. The animals were maintained under standard laboratory conditions at 22 ± 2°C with 12-h light/dark cycles and humidity at 55 ± 5%.

### Induction of diabetes

Diabetes was induced in overnight fasted rats by a single intraperitoneal injection of a freshly prepared solution of streptozotocin (STZ, Sigma, USA) in citrate buffer (0.1 M, pH 4.5) at a dosage of 50 mg/kg body weight (b.wt.). Diabetes was confirmed by stable hyperglycemia (>18 mmol/l) in the tail blood glucose after five days of STZ injection using a portable glucometer (Accu-Chek, Roche, Germany).

### Study design

The dose of kolaviron (100 mg/kg) was chosen based on our preliminary investigation. 100 mg/kg kolaviron was a more effective dose among the doses (100 and 200 mg/kg) investigated in our preliminary study. The animals were divided into 4 groups (n = 10 per group): Normal control (NC group), Kolaviron treated normal control (NC + KV), diabetic control (DM group), and kolaviron treated-diabetic group (KV + DM group). Kolaviron (100 mg/kg b.wt.), dissolved in dimethylsulphoxide (DMSO) was administered by gastric gavage 5 times a week. Control rats also received DMSO as a vehicle. At the end of the treatment period, the rats were weighed and then anaesthetized with an intraperitoneal injection of sodium pentobarbital (60 mg/kg). Blood glucose was measured in 4 hours-fasted animals (usually between 10 am and 2 pm). Blood samples were collected from the abdominal aorta into glucose tubes (containing sodium fluoride/potassium oxalate), EDTA-containing tubes and serum clot activator tubes. Blood samples were centrifuged at 4000 g for 10 min at 4°C. Aliquot of the supernatant was stored at - 80°C for plasma glucose determination while other biochemical analysis was carried out on the serum. The liver was dissected out, rinsed in cold phosphate buffered saline (10 mM pH 7.2), blotted on filter paper and weighed. Liver homogenate was prepared in phosphate buffered saline (10 mM pH 7.2), centrifuged at 15000 rpm for 10 min at 4°C.

### Liquid chromatography-mass spectrometry (LC-MS) analysis of *Garcinia kola* seed extract

LC-MS was performed on a Dionex HPLC system (Dionex Softron, Germering, Germany) equipped with a binary solvent manager and autosampler coupled to a Brucker ESI Q-TOF mass spectrometer (Bruker Daltonik GmbH, Germany). Kolaviron was separated by reversed phase chromatography on a Thermo Fischer Scientific C18 column 5 μm; 4.6 × 150 mm (Bellefonte, USA) using gradient elution with 0.1% formic acid in water (solvent A) and acetonitrile (solvent B) as solvent at a flow rate of 1.0 ml min^-1^, an injection volume of 10 μl and an oven temperature of 30°C. MS spectra were acquired in negative mode using the full scan and auto MS/MS (collision energy 25 eV) scan modes with dual spray for reference mass solution. Electrospray voltage was set to +3500 V. Dry gas flow was set to 9 l min^-1^ with a temperature of 300°C and nebulizer gas pressure was set to 35 psi.

### Analysis of glucose and liver dysfunction biomarkers

Plasma glucose, levels of aspartate transaminase (AST) and alanine transaminase (ALT) in the serum were analyzed with diagnostic kits in an automated clinical chemistry analyzer (Medical Cooperation, Bedford, MA, USA).

### Insulin estimation

Plasma insulin was estimated by radioimmunoassay (RIA) according to the protocol supplied by Merck Millipore (Millipore, Cooperation, MA, USA). Separate tubes containing 100 *μ*L and 200 *μ*L of assay buffer, 100 *μ*L of plasma samples or standards were mixed with100 *μ*L ^125^I insulin tracer and 100 *μ*L of primary antibodies. The mixture was incubated overnight at 4°C. This was followed by the addition of 1 mL of precipitating agent and incubation for 20 minutes at 4°C. Again, samples were centrifuged at 4000 g for 30 min at 4°C and the supernatant was aspirated. The tubes were subjected to radioactive counting using a ^125^I gamma counter.

### Analysis of inflammatory biomarkers

The levels of 4 inflammatory markers including interleukin (IL)-1β, IL-6, tumour necrosis factor (TNF)-α and monocyte chemotactic protein (MCP-1) were measured in the tissue lysates from all the rats. This was done using Bio-plex Pro™ magnetic bead-based assays (Bio-Rad Laboratories, Hercules, USA) on the Bio-plex® platform (Bio-Rad), according to the manufacturer’s instructions. Following previous optimization, samples were evaluated undiluted, in a blinded manner. Bio-Plex Manager™ software, version 6.0 was used for bead acquisition and analysis.

### Statistical analysis

Data were analyzed using one-way analysis of variance and expressed as mean ± standard deviation. Statistical analyses were performed using Graph Pad Prism version 6.02, for windows (Graph Pad software, San Diego, CA). Differences were considered significant at P < 0.05.

## Results

### Kolaviron treatment lowers blood glucose, prevented loss of body weight and liver hypertrophy in diabetic rats

Effect of kolaviron administration on blood glucose, liver and body weight in STZ-induced diabetic and normoglycemic rats is shown in Table 1. Six weeks after diabetes confirmation, the random blood glucose concentration (mmol/l) in the diabetic and control group was 28.19 ± 2.25 and 9.93 ± 0.51 respectively. The blood glucose concentration for the normal control rats plus KV was 8.91 ± 0.6 and the diabetes mellitus plus kolaviron group was 17.35 ± 2.36. In addition to elevated blood glucose, diabetic rats had decreased mean body weight compared to normal control while treatment with kolaviron for 6 weeks significantly lowered blood glucose and ameliorated the body weight loss when compared to the untreated diabetic group, although body weight was still significantly lower in comparison with normal control. Diabetes caused an increase in relative liver weight (expressed as % body weight) in rats while treatment of diabetic rats with kolaviron significantly restored liver weight to near normal. STZ diabetic rats exhibited impaired insulin release while KV treatment of diabetic rats significantly increased plasma insulin levels compared with untreated rats.

**Table 1:** Effect of kolaviron administration on plasma glucose, insulin, liver weight and body weight in STZ-induced diabetic and normoglycemic rats

**Parameters**	**NC**	**NC + KV**	**DM**	**DM + KV**
**Glucose (mmol/l)**	9.93 ± 0.51	8.91 ± 0.6^a,b^	28.19 ± 2.25^a^	17.35 ±2.36^a,b^
**Insulin (ng/ml)**	7.99 ± 1.93	7.69 ± 1.94	0.30 ± 0.13^a,b^	0.79 ± 0.28^a,b^
**Body weight change (g)**	+99.59	+101.12	−9.38^a^	+45.38^a,b^
**Relative liver weight (g)**	3.22 ± 0.15	3.38 ± 0.07	4.53 ± 0.16^a^	3.99 ± 0.15^a,b^

Data are presented as mean ± S.D Values. ^a^p < 0.05 vs. normal control. ^b^p < 0.05 vs. untreated diabetes. NC; Normal control, NC + KV; Normal control treated with kolaviron, DM; untreated diabetic rats, DM + KV; Diabetic rats treated with kolaviron.

### Kolaviron lowers serum levels of hepatic enzymes in STZ-induced diabetic rats

**Figure 1:**
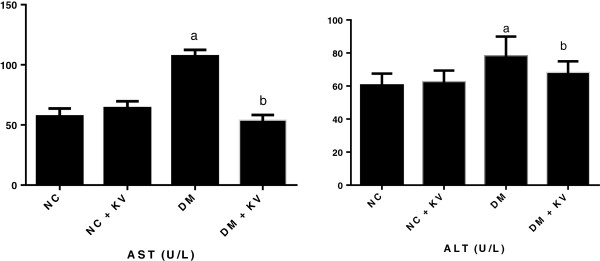
**Effect of kolaviron administration on serum levels of hepatic enzymes in diabetic and normoglycemic rats.** Data are presented as mean ± S.D values. ^a^p < 0.05 vs. normal control. ^b^p < 0.05 vs. untreated diabetes. NC; Normal control, NC + KV; Normal control treated with kolaviron, DM; untreated diabetic rats, DM + KV; Diabetic rats treated with kolaviron.

Figure 1 indicates results of kolaviron administration on serum levels of hepatic enzymes in STZ**-**induced diabetic rats. In the diabetic group, serum levels of liver damage biomarkers; ALT (77.96 ± 11.9) and AST (107 ± 5.43) were elevated compared with normal controls viz: 60.37 ± 7.20 and 57.12 ± 6.63 respectively. Kolaviron administration to diabetic rats significantly reduced serum levels of ALT (67.9 ± 6.94) and AST (53.38 ± 4.93) when compared to diabetic control.

### Kolaviron ameliorated hyperglycemia-mediated increase in the levels of proinflammatory proteins in the liver of diabetic rats

**Figure 2:**
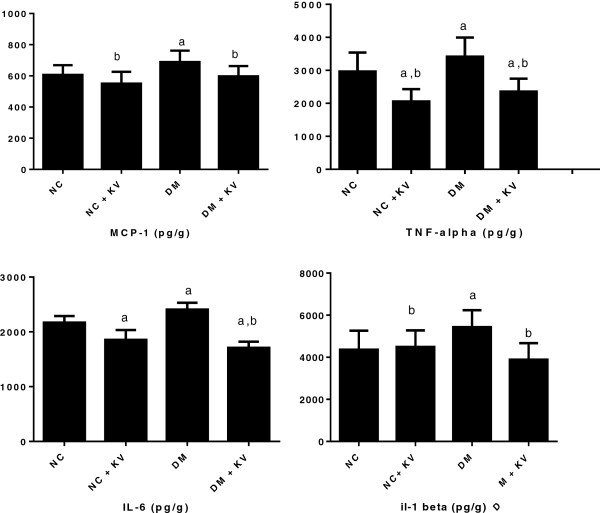
**Effects of kolaviron on levels of MCP-1, IL-1β, TNF-α and IL-6 in the liver of normal and diabetic rats.** Data are presented as mean pg/g wet tissue ± S.D. ^a^p < 0.05 vs. normal control. ^b^p < 0.05 vs. untreated diabetes. NC; Normal control, NC + KV; Normal control treated with kolaviron, DM; untreated diabetic rats, DM + KV; Diabetic rats treated with kolaviron.

The effect of kolaviron on interleukin (IL)-1β, IL-6, tumour necrosis factor (TNF-**α**) and monocyte chemotactic protein (MCP-1) is illustrated in Figure 2. The concentration of proinflammatory cytokines were significantly increased in the liver of diabetic rats when compared with the control rats. Lowered levels of MCP-1 and IL-1β were detected in the liver of kolaviron treated diabetic rats compared to the untreated diabetic group. Administration of kolaviron to diabetic rats 5 times a week for 6 weeks also significantly reduced IL-6 and TNF-**α** when compared with both normal control and diabetic rats. Kolaviron also lowered serum levels of TNF-**α** and IL-6 in normal rats.

### Liquid chromatography-mass spectrometry (LC-MS) analysis of *Garcinia kola* seed extract

**Figure 3:**
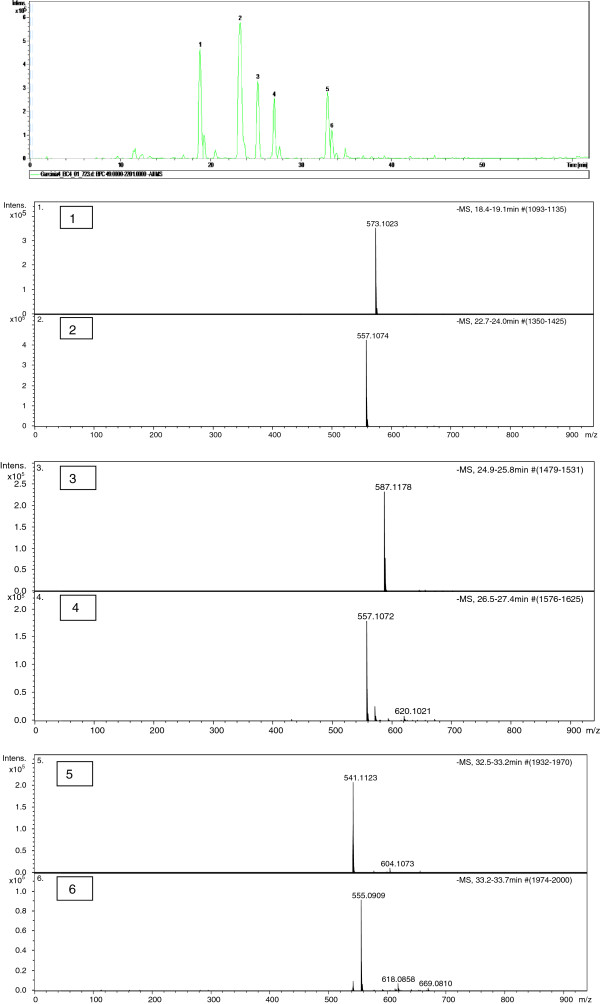
**Mass Spectra of kolaviron.** (1) *Garcinia* biflavonoid 2 (C_30_H_22_O_12_, *m/z* 573.1023); (2) *Garcinia* biflavonoid 1 (C_30_H_22_O_11_, *m/z* 557.1074); (3) Kolaflavanone (C_31_H_24_O_12_, *m/z* 587.1178); (4) *Garcinia* biflavonoid 1 (C_30_H_22_O_11_, *m/z* 557.1074); (5) X (C_30_H_22_O_10_, 541.1123), deduced to be binaringenin); (6) Kolaflavone (C_30_H_21_O_11_, *m/z* 555.0909).

**Figure 4:**
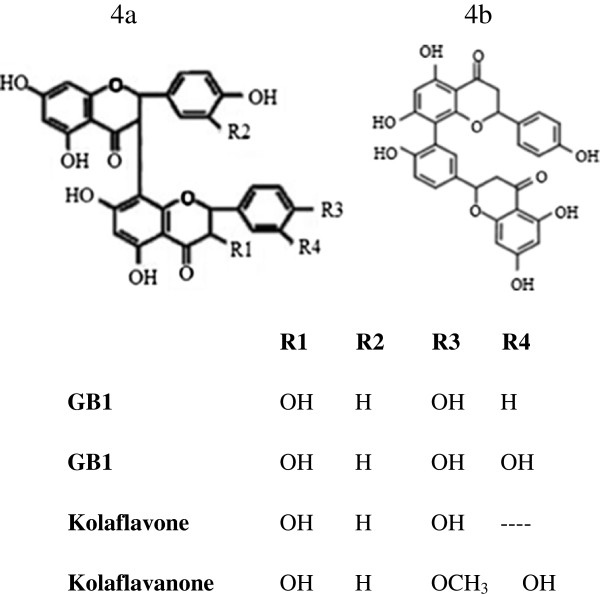
**Chemical structure of *Garcinia *biflavonoid complex.** (Kolaviron) containing *Garcinia* biflavonoid GB-1(3″,4′,4‴,5,5″,7,7″-heptahydroxy-3,8″ biflavanone), GB-2 (3″,4′,4‴,5,5″,5‴,7,7″-octa-hydroxy-3,8″- biflavanone), and kolaflavanone (3″,4′,4‴,5,5″,5‴,7,7″ octahydroxy-4‴-methoxy-3,8″-biflavanone) is confirmed (Figure 4a) while binaringenin (Figure 4b) is presumed to be an additional compound in kolaviron based on ESI-MS/MS result.

In the negative-ion, the ESI-MS analysis of kolaviron shows molecular ion peaks [M-H]^-^ at (1) *m/z* 573.1023*,* (2) *m/z* 557.1074*,* (3) *m/z* 587.1178*,* (4) *m/z* 557.1074 (5) *m/z* 541.1123 and (6) *m/z* 555.0909 (Figure 3). Based on the calculated molecular formula, the presence of *Garcinia* biflavonoid 2 (*m/z* 573.1023 = C_30_H_22_O_12_), *Garcinia* biflavonoid 1(*m/z* 557.1074, 557.1072 = C_30_H_22_O_11_), kolaflavanone (*m/z* 587.1178 = C_31_H_24_O_12_) and kolaflavones (*m/z* 555.0909 = C_30_H_21_O_11_), previously reported in literature as components of kolaviron [16] were confirmed (Figure 4a). The mass spectrum of peak 5 shows the ion peak [M-H]^-^ at *m/z* 541.1123 with the formula C_30_H_22_O_10_. On the basis of this data and database searching, the structure of this compound (peak 5) was deduced to be a binaringenin (Figure 4b), a biflavanone commonly found in *Garcinia* species [23] To our knowledge, this is the first report of a new biflavonoid in kolaviron (a *Garcinia* biflavonoid complex).

## Discussion

Limitations of the currently used drugs on glycemic regulation have raised the need for the development of new drugs which can act as alternative and/or complementary therapy. Interest in natural plant products as anti-diabetic agents has increased over the years due to their low side effects and multidimensional mode of action [24]. Kolaviron a natural compound from the bitter kola seed, containing a complex mixture of *Garcinia* biflavonoid, GBI, GB2 and kolaflavones has been demonstrated for its, hypoglycemic, antioxidative, anti-inflammatory and antigenotoxic effects [19,25].

The concept that chronic low grade inflammation is important in the development and progression of diabetes and its associated complication is not new. Prolonged hyperglycemia can generate an inflammatory state leading to an increment in cytokine production and pancreatic beta cell destruction [26]. Anti-inflammatory agents have been documented to be beneficial in diabetes. Among these are curcumin and its derivative [27,28], resveratrol [29] and cannabidiol [30]. The damaging effect of inflammatory molecules in diabetes can be mediated through its interaction with receptors and activation of signaling pathways which exacerbate the disease state [31]. Due to the existing association between chronic inflammation and diabetic complications including liver injury, identification of therapeutic targets that is able to specifically downregulate proinflammatory responses and mediators could be a promising strategy in the management of diabetes mellitus. It is noteworthy that our study is the first to investigate the effect of kolaviron on inflammatory mediators in diabetes.

Regulation of blood glucose either in the fasting state and/or postprandially is an important factor in diabetic therapy. Sustained glycemic control decreases the risk of developing microvascular and macrovascular complications [32,33]. The marked reduction of blood glucose in this study following KV treatment is in line with previous studies demonstrating its hypoglycemic effects [34]. Although kolaviron significantly increased the plasma insulin levels in diabetic rats, the magnitude of increase is lower compared to the corresponding effect on blood glucose. The mechanisms of hypoglycemic effect of kolaviron might be due to the combination of its stimulating action on the pancreatic β cells to release insulin and also an insulin independent effect and extrapancreatic action which involves glucose utilization in extrahepatic tissues [35,36]. Furthermore, the hypoglycemic effect of flavonoids can be mediated through an increase in hepatic glucose storage by stimulating the action of glycolytic and glycogenic enzymes or by inhibiting glucose-6-phosphatase. This consequently results in the uptake of glucose into cells and the reduction in the blood glucose level through the upregulation of glycogen formation, downregulation of the rate of glycogen breakdown, and glucose synthesis [37-39].

It has been shown that absolute or relative insulin deficiency coupled with decreased ATP production accounts for low protein synthesis [40]. The decreased mean body weight in diabetic rats could be an indication of excessive breakdown of structural proteins in an attempt to compensate for low availability of carbohydrate as an energy source [41]. The ability of kolaviron to protect against weight loss might be due to its glucose lowering effect. Liver hypertrophy (increased liver weight) observed in diabetic rats may be due to hypoinsulinemia- induced increased triglycerides accumulation in the liver as alternative glucose precursors since liver glycogen is usually depleted in STZ-induced diabetic rats [42]. Liver weight (expressed as a percentage of body weight) was significantly lower in kolaviron treated rats. The ability of kolaviron to restore liver glycogen levels may partly explain its beneficial effect on liver hypertrophy in diabetic rats. It was reported in a previous that kolaviron inhibited microsomal glucose-6-phosphatase in STZ diabetic rats [34]. The inhibition of glucose-6 phosphatase by kolaviron can increase hepatic glucose-6 phosphate which serves as substrate for glycogen synthesis thereby resulting in upregulation of hepatic glycogen levels.

Amino transferases, aspartate transaminase (AST) and alanine transaminase (ALT) catalyse amino transfer reactions and are used as markers of hepatic injury [43]. Deleterious effect of hyperglycemia in the liver of diabetic rats observed in the present study is evidenced by serum elevation of liver damage biomarkers. The hepatoprotective effect of kolaviron is demonstrated by the significant reduction of serum levels of ALT and AST in the diabetic treated rats.

Inflammation has been reported to cause direct organ damage in diabetic rats [44,45] and humans [46]. Increased levels of pro-inflammatory cytokines MCP-1, IL-1β, IL-6, IL-18 and TNF-α have been reported in diabetes [47,48]. In this study, upregulated levels of these pro-inflamatory proteins were observed in the liver of STZ-induced diabetes rats.

MCP-1 is a chemo-attractant which promotes monocyte and macrophage migration and activation at the site of injury. Over-expression of MCP-1 exerts various damaging effects via increased production of superoxide radicals from macrophages, release of lysosomal enzymes, cytokines, growth factors and cellular adhesion molecules [49,50]. Animal and human studies have also shown a correlation between blood and hepatic levels of MCP-1 and the extent of inflammation [51,52]. Considering the role of macrophages in perpetuating hepatic inflammation, reduction of MCP-1 levels may be another mechanism by which kolaviron mediates its protective effect in the liver of diabetic rats.

Hepatic infiltrating macrophages and Kupffer cells are sources of pro-inflammatory cytokines such as TNF-α, IL-1 and IL-6 in the liver [53]. Our study shows upregulated levels of these inflammatory proteins in a diabetic state while kolaviron treatment notably reduced hepatic levels of IL-1β, IL-6 and TNF-α in diabetic rat. The suppressing effect of kolaviron on serum levels of IL-1β has been demonstrated in chemically-induced inflammation of the colon [19]. IL-1β induces the expression of various genes encoding oxidants, cytokines, chemokines, growth factors and adhesion molecules whose promoter region are monitored through interactions with transcription factor, NFκB [54-56]. IL-1β inhibits β- cell function and promotes Fas-triggered apoptosis in part by NF-κB [57]. In a GK rat model of type 2 diabetes, treatment with IL-1 receptor antagonist (IL-1Ra) reduced islet mRNA expression of a number of inflammatory factors which includes: IL-1β, IL-6, TNF-α, MCP-1 and MIP-1α [58]. Possible mechanism by which kolaviron elicit its liver protective and anti-inflammatory effect in diabetic rats could be by a direct reduction of macrophage infiltration and/or by repressing NF-κB activation.

TNF-α is one of the major cytokines upregulated in diabetic liver which can promote the activation of NF-κB through interaction with the TNF-α receptor resulting in liver inflammation and apoptosis [55]. The involvement of TNF in alcoholic hepatitis, viral hepatitis and ischemia/reperfusion liver injury has also been documented [59]. Our study revealed that Kolaviron treatment abrogated hyperglycemia induced increase in the hepatic concentration of TNF-α. There is a report that kolaviron (KV) shows inhibitory action on prostaglandin E_2_ and TNF-α production in macrophage-like cell line [60]. Kolaviron also downregulates iNOS and COX-2 expression in the liver of dimethyl nitrosamine (DMN)-treated rat via the inhibition of DNA binding activity of NF-κB [61]. The anti inflammatory and hepatoprotective effect observed in our study might be mediated via inhibition of transcription factor NF-κB, a key regulator of inflammatory process.

Although the results of our study demonstrated the beneficial effects of kolaviron on inflammatory response and hepatic injury in the liver of diabetic rats, future studies can address other possible mechanisms of action of kolaviron and the underlying molecular targets of *Garcinia* biflavonoid complex in diabetes. Further investigation may also be necessary for complete elucidation of the structure of kolaviron.

## Conclusion

In summary, our study revealed that kolaviron treatment ameliorated hyperglycemia-induced increase in the levels of proinflammatory cytokines and chemokine in rat’s liver and may therefore act as a useful agent in retarding the progression of diabetic liver complications. Another important outcome of this study is the discovery of a new compound from kolaviron. This new compound along with previously identified compounds of kolaviron may partly explain its beneficial effects in diabetes.

## Competing interests

The authors declare that they have no competing interests.

## Author’s contributions

ORA was responsible for the conception and design, carried out all experiment, performed data analysis and drafted the manuscript. NNC collaborated in the antiinflammatory studies and made contribution to the revision of the manuscript. NLB and OOO made contribution to the conception and revised the manuscript critically for intellectual content. All authors read and approved the final manuscript.

## Pre-publication history

The pre-publication history for this paper can be accessed here:

http://www.biomedcentral.com/1472-6882/13/363/prepub
